# Protein quality control systems in neurodegeneration – culprits, mitigators, and solutions?

**DOI:** 10.3389/fneur.2025.1604076

**Published:** 2025-09-03

**Authors:** Aaron Ciechanover, Ido Livneh

**Affiliations:** ^1^The Rappaport Technion Integrated Cancer Center (R-TICC) and The Rappaport Faculty of Medicine and Research Institute, Technion-Israel Institute of Technology, Haifa, Israel; ^2^Institute of Pathology and Cytology, Rambam Health Care Campus, Haifa, Israel

**Keywords:** ubiquitin-proteasome system, protein quality control (PQC), neurodegenarative disease, autophagy, protein aggregates

## Abstract

A key hallmark of neurodegenerative diseases (NDDs) is the formation of neurotoxic protein aggregates, which are considered to reflect inadequate protein quality control (PQC). In agreement with this fundamental pathophysiologic characteristic, the two main cellular systems responsible for cellular protein removal – the ubiquitin-proteasome system (UPS) and autophagy – have been extensively studied in the context of NDD. The involvement of these proteolytic machineries was interpreted in different ways – some pointed them as dysfunctional systems that may underlie pathogenesis, while others suggested they fulfill protective roles which delay the clinical presentation of these diseases. Perhaps not surprisingly, the growing body of knowledge concerning the different types of NDD portrays a more complex picture, and no distinct generalization can be made regarding the contribution of either the neurotoxic protein substrate(s) or proteolytic system(s) to the development of NDD. For instance, in Parkinson’s disease, the toxic aggregation of *α*-synuclein, Parkinson’s canonical culprit protein, can stem from seemingly unrelated events. Among them, alterations in *α*-synuclein itself, a mutation in Parkin – an E3 ubiquitin ligase targeting proteins and organelles to proteasomal and lysosomal degradation, respectively, as well as a mutation in LRRK2 – a kinase postulated to be linked with α-synuclein through their common removal by chaperone-mediated autophagy. Also, in amyotrophic lateral sclerosis (ALS) and frontotemporal lobar degeneration (FTLD), the toxic aggregation of one protein – TDP-43 – can result from defects in other proteins, some of which are related to proteostasis, such as the shuttle protein Optineurin and the E3 ubiquitin ligase VCP. In contrast, ALS and FTLD demonstrate how common abnormalities leading to neurotoxic aggregate formation, may present clinically in profoundly different ways, from motor dysfunction to behavioral changes. In Alzheimer’s Disease, the leading cause for dementia, rare cases were linked directly with PQC as they are caused by a mutation in one of the genes encoding ubiquitin itself, while the majority of cases were not directly linked to components of the two main proteolytic systems. All-in-all, the UPS and autophagy are heavily intertwined with NDD, either as part of the problem or as mitigating factors, and hopefully – as platforms for future therapeutics. In this review, we shall dissect NDDs from the perspective of protein turnover pathways, aiming to track both common and unique patterns of PQC failure in this group of diseases, which differ significantly from one another both in their clinical manifestations and affected anatomic regions, yet share the common trait of abnormal protein accumulation. We shall review some of the mechanistic understandings concerning protein aggregation in NDDs, describing the interactions of aggregated proteins with the UPS and autophagy, discuss recent controversies around the protein aggregates’ hypothesis, and point to implications for developing therapeutic strategies.

## Introduction

### The ubiquitin-proteasome system – a selective protein predator

**Figure 1:**
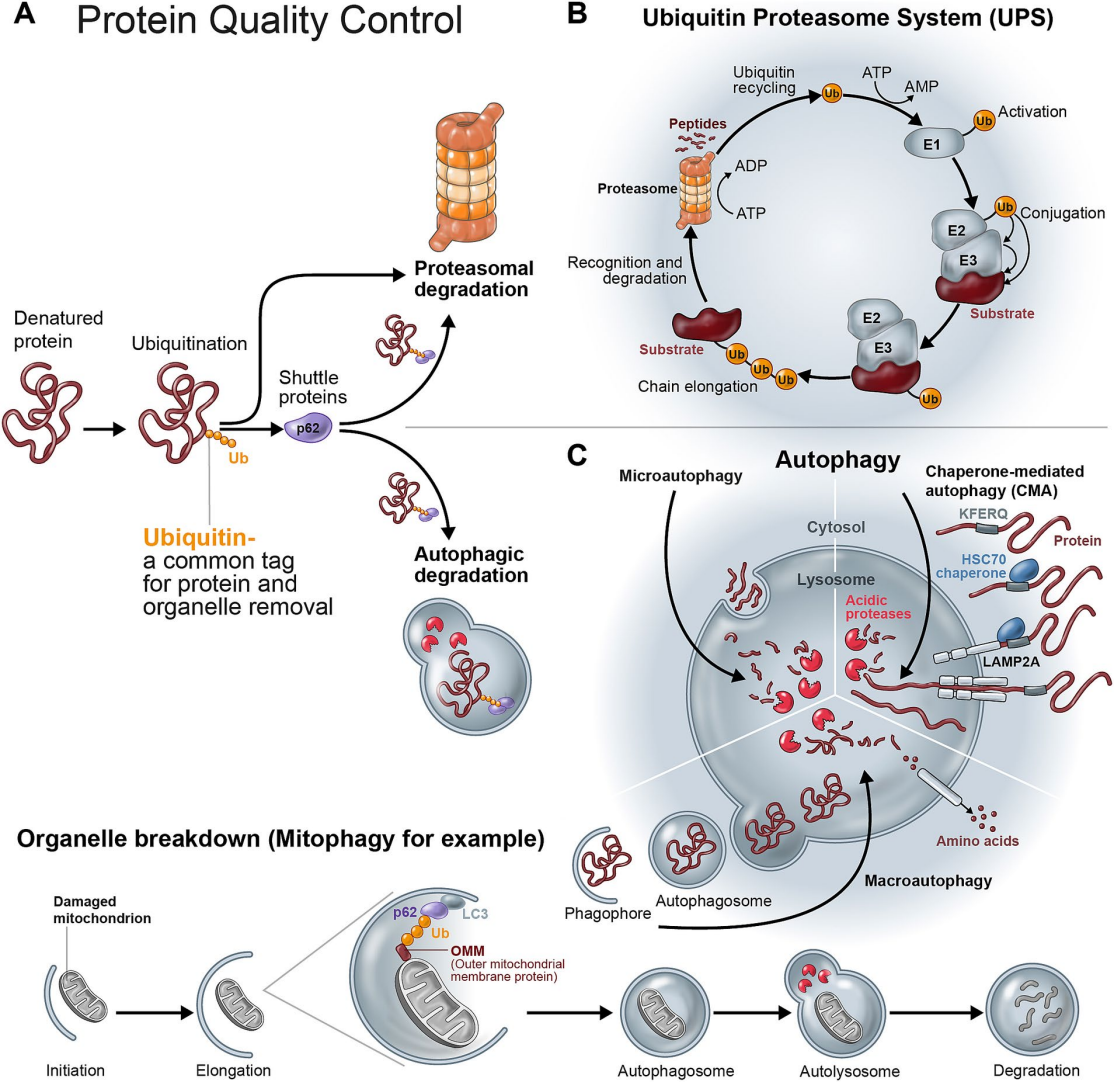
Protein quality control by the ubiquitin-proteasome system and the autophagy-lysosome pathway. **(A)** Ubiquitin serves as a recognition signal that marks proteins destined for degradation. These proteins are either misfolded/denatured or otherwise damaged proteins, or proteins that completed their function (e.g., cell cycle regulators). Ubiquitinated proteins can be recognized by inherent proteasomal ubiquitin receptors, and/or via soluble ubiquitin receptors which serve as shuttle proteins. These shuttle proteins (e.g., SQSTM1/p62) can target ubiquitinated proteins to either proteasomal or autophagic degradation. **(B)** The removal of proteins by the ubiquitin-proteasome system begins with the ATP-dependent activation of ubiquitin molecules by the ubiquitin-activating enzyme E1. Ubiquitin moieties are next transferred to a ubiquitin-conjugating enzyme (also known as ubiquitin-carrier protein), or E2. A third enzyme, E3 or ubiquitin ligase, brings the ubiquitin-carrying E2 and the substrate protein to the proximity that facilitates the transfer of the ubiquitin moiety to a lysine residue in the substrate. E3 ligases largelly belong to one of two classes: RING (Really INteresting Genes) E3s catalyze ubiquitin transfer from the E2 directly to the substrate, while HECT (Homologous to the E6-AP Carboxyl Terminus) domain E3s first bind the ubiquitin molecule, prior to its conjugation to the substrate. In both cases, a ubiquitin chain is elongated through the conjugation of additional ubiquitin moieties on lysine residues of a previously attached ubiquitin. **(C)** Protein degradation via autophagy is facilitated by deliverying substrates into the lysosome, where they are digested by acidic proteases. There are 3 different mechanisms through which proteins are delivered into the lysosome for their degradation: (1) Microautophagy, in which proteins are swallowed through invagination of cytoplasmic droplets into the lysosome. (2) Chaperone-mediated autophagy, where certain proteins are recognized by chaperones and are inserted into the lysosome via channels located on the lysosomal membrane. (3) Macroautophagy, under which proteins, voxels of cytoplasm, and/or organelles are engulfed by a growing membrane to eventually form an autophagosome, which subsequently fuses with the lysosome (producing an autolysosome) thereby injecting its cargo into the acidic and catalytic environment of the lysosome. Organelles such as the mitochondria are also destined for macroautophagy via ubiquitination (e.g., of outer mitochondrial proteins), which is recognized by shuttle proteins such as p62 that also binds to the autophagic receptor LC3, thereby securing the binding of its cargo to the forming autophagosome which will later be delivered into the lysosome.

The ubiquitin-proteasome system (UPS) is one of the two major proteolytic systems in eukaryotes, alongside autophagy, and is responsible for the majority of selective protein removal (Figure 1A). Similar, other systems for protein degradation were identified in certain prokaryotes (1, 2). The process leading to degradation of a protein substrate by the UPS can be largely divided into two sequential steps: (i) recognition of the substrate and its covalent marking by ubiquitin, mediated by a specific ubiquitin ligase (E3); and (ii) degradation of the tagged protein by the 26S proteasome with release of reusable ubiquitin (Figure 1B). The first step, i.e., ubiquitination, is by itself comprised of a sequence of biochemical reactions, only the last of which involves the attachment of a ubiquitin molecule to the protein substrate. First, one of two ubiquitin-activating enzymes, or E1, renders a ubiquitin moiety available for binding by activation of its C-terminal glycine in an ATP-dependent manner. The activated ubiquitin is transferred to an E2, or ubiquitin-carrier enzyme, which will generate a ternary complex with a protein substrate bound to its cognate E3 ligase. Finally, the E3 will transfer the ubiquitin moiety to the protein substrate, or ligate it to a previously attached ubiquitin – thereby forming (or elongating) a ubiquitin chain. Some E3s transfer ubiquitin directly from the carrier E2 to the substrate/elongating chain (RING E3s), while others first bind ubiquitin prior to its further ligation to the target (HECT E3s). Not surprisingly, given the vast number of E3s encoded by the human genome, a hybrid type of E3 ligases was described as well (3).

Importantly, E3 ubiquitin ligases are the components within the UPS providing it with selectivity toward its protein substrates. While ubiquitin itself is a degradation signal common to all proteasomal substrates, the proteasome recognizes substrates destined for degradation based on their ubiquitination rather than their specific identity. At the same time, in order to facilitate concerted protein degradation in response to a specific condition such as ER stress, or to meet a discrete cellular requirement such as the removal of misfolded proteins, the UPS employ some “universal” E3 conjugating machineries, such as the ER Hrd1 ligase complex (4) and CHIP (carboxyl terminus of Hsc70-interacting protein) – the chaperone-associated ligase (5).

The ubiquitination process is reversible, and recycling of ubiquitin molecules occurs via its removal from ubiquitinated substrates, i.e., deubiquitination. This reaction is carried out by deubiquitinating enzymes (DUBs) along proteasomal degradation of the target proteins, which allows reuse of most of the ubiquitin molecules. Ubiquitin moieties can also be removed from proteins that have not yet been recognized by the proteasome, representing what can be regarded as a rescuing mechanism of proteins that – for example – have been ubiquitinated by mistake, or that following their tagging have been refolded to their native form. Deubiquitination therefore serves as an additional layer of regulation of protein removal, balancing the activity of E3 ubiquitin ligases (6).

Protein ubiquitination and subsequent degradation are constantly carried out in cells, serving three main functions: (i) timely removal of native proteins that have completed their function, such as cell cycle regulators and transcription factors. Some proteins also must be signaled for their targeting by phosphorylation or oxidation, for example, in order for the UPS to recognize them (7). An interesting question is why these proteins are destroyed rather than being sequestered or inactivated by a posttranslational modifications, which will be “cheaper” energy- and metabolic-wise. We assume that the reason is that degradation is irreversible, thus preserving the directionality of essential biological processes such as DNA replication; (ii) maintenance of protein quality control (PQC) by the removal of “abnormal” (e.g., mutated, denatured, oxidized, aggregated) proteins that their accumulation maybe harmful to the cell; and (iii) removal of proteins under stress (e.g., starvation, heat shock, hypoxia) in order to re-allocate nutritional/energy resources to cope with the stress (8). It should be noted that the three main reasons for removal of a protein by the UPS, the substrates are tagged in a highly selective and specific manner. All-in-all, selective protein degradation by the UPS ensures the organism’s ability to maintain the integrity of the proteome and to respond to the everchanging cellular cues.

### Autophagy – massive and bulky protein removal

Autophagy, Greek for “self-eating,” refers to the degradation of proteins in the lysosome, while the substrates destined for breakdown can reach the acidic organelle via several different ways (Figure 1C). In the classic autophagic pathway, also termed macro-autophagy, a membrane bound autophagosome is engulfing cytosolic contents, be it soluble proteins or whole organelles (e.g., mitochondria, ER, peroxisomes), and subsequently brings its cargo into the lysosome via vesicular membrane fusion. The proteins that are “swallowed” by the forming autophagosome may be targeted to it by their ubiquitination and subsequent identification by a ubiquitin-binding shuttle protein such as sequestosome 1 (SQSTM1, also known as p62) which will deliver the substrate to the autophagosome via binding to the autophagic membrane-bound receptor – Microtubule-associated protein 1 light chain 3 – commonly referred to simply as LC3. At the same time, a non-targeted engulfment of bystander proteins localized to the cytoplasmic “voxel” around which the autophagosome is forming, is inevitable. Another type of autophagy is termed microautophagy, in which the lysosomal membrane – via invagination – “pinches” small volumes of cytoplasm and their resident proteins, releasing them into the lysosomal lumen. While microautophagy is largely considered to be non-selective, a third type of autophagic degradation, namely chaperone-mediated autophagy (CMA), involves selective targeting of individual proteins for lysosomal degradation (9). Recognition of these proteins is mediated by a specific internal motif, the consensus of which is made of the amino acids KFERQ (10).

While degradation by the proteasome is virtually limited to ubiquitinated proteins, autophagic degradation is inherently less selective, as mentioned above. Nevertheless, the removal of organelles, membrane receptors, as well as some soluble proteins by autophagy, was shown to be selective, and in many of these cases – targeting for autophagic breakdown was in fact found to be ubiquitin-dependent. Time-wise, the ubiquitin system is first to respond to a cellular cue, whereas autophagy, is slower to respond in order to postpone as much as possible degradation of normal functioning proteins and the time necessary for generation of the autophagosomes. While the differences (and overlap) between signals destining proteins for removal by either the UPS or autophagy were discussed elsewhere (11–13), of note is the reciprocal relationship between the two, demonstrated by the compensatory activity of one following inhibition of the other. Accordingly, a discussion concerning inadequate PQC and/or pathologic protein aggregation should take into account possible dysregulation of either system.

### Trafficking proteins to their destiny: shuttling tagged proteins to the proteasome and autophagy

As described above, the recognition of a substrate protein that was destined for degradation via its conjugation with ubiquitin molecule(s), and its subsequent delivery to the forming autophagosome, is inherent to selective macroautophagy. Notably, while the proteasome contains internal subunits that function as receptors for the ubiquitination signal, a recognition which is sufficient for substrate binding and degradation, some ubiquitinated substrates can be recognized, bound, and delivered to the proteasome by soluble shuttling proteins. These specialized traffickers usually harbor two recognition elements: (i) binding ubiquitinated proteins via a ubiquitin-associated domain (UBA), and (ii) recognizing the proteasome in order to target the ubiquitinated substrate for degradation. p62, for instance, recognizes the proteasome in order to deliver ubiquitinated substrate for degradation via its Phox and Bem1 (PB1) domain.

Similar principles apply in the context of selective macroautophagy, where a set of adaptor proteins facilitates the targeted delivery of ubiquitinated substrates to the autophagosome. These include p62 itself, NBR1 (Neighbor of BRCA1 gene 1), NDP52 (Nuclear Dot Protein 52 kDa), optineurin, and Tax1BP1 (Tax1 binding protein 1), which bind polyubiquitinated cargo through their ubiquitin-binding domains and tether it to the autophagosomal membrane via LC3-interacting regions (LIR). This recognition scheme allows for specific substrate targeting, analogous to proteasomal shuttling, but directed toward autophagic degradation. Many of these adaptors are also substrates of autophagy and are degraded together with their cargo, highlighting their transient role in cargo recruitment and turnover. These adaptor-mediated pathways contribute to the broader understanding of macroautophagy as a process capable of substrates’ selection, in addition to its established role in bulk degradation (9). Intriguingly, p62 was shown to reside within protein aggregates in NDDs such as Alzheimer’s neurofibrillary tangles and Parkinson’s Lewy bodies, and its association with such inclusions is mediated by its ability to bind ubiquitinated proteins (14).

Specifically, p62 can also shuttle substrate proteins for autophagic degradation through binding to LC3 on growing autophagosomes via its LIR. Moreover, p62’s autophagic substrates include the proteasome itself, which following its delivery is degraded in the lysosome (15). In addition for targeting the proteasome for its autophagic removal (16, 17), p62 can also alter proteasomal subcellular compartmentation in response to stress. During amino acids starvation, it assists in translocation of the proteasome from its main storage in the nucleus to the cytosol where it degrades cellular proteins, providing the cell with the amino acids required for its survival under the shortage (18, 19). This intricate relationship between a shuttle protein and the two major proteolytic systems highlights the key role of spatial regulation in PQC.

### Neurodegenerative diseases – how dysregulation of protein removal derails us?

Neurodegenerative diseases (NDD) are a group of diverse neurological pathologies, largely characterized by progressive loss of neurons due to the toxic effect(s) of aggregated misfolded proteins. These proteins vary between the different conditions, yet some of them can be found in more than a single NDD. While the exact pathogenesis underlying most NDDs is yet to be fully understood, as do the mechanism(s) through which NDD-related proteins exert their neurotoxic effect, it is accepted that accumulation of the aggregated proteins probably lies at the core of the pathology of many NDDs (20). Further, it was shown that aggregates may have a protective role, at least at the initial phase of the disease which is due to their sequestration away from critical cellular machineries (see below). Since the pathologic sequence of events underlying many of these conditions involves failure of the PQC mechanism(s), and the aggregated proteins are largely considered as the culprits, NDDs are also referred to as proteinopathies. Notably, unlike carbohydrate errors of metabolism, for instance, which are presenting early in life, NDD usually presents itself clinically at an advanced age. With some exceptions, even familial cases of NDD that are referred to as early-onset, become clinically apparent during adulthood. The fact that even NDD-causing mutations take decades to manifest clinically, points out to a multifactorial pathogenesis, environmental contribution, and an amazingly high affected tissue reserve. Most cases of NDD are age-related, and the lifetime risk for Alzheimer’s dementia, for example, is ~10–20%, starting at the 7^th^ decade of life with a substantial prevalence in people who are >85-year-old. Independent mechanistic studies suggest that the cellular systems responsible for PQC, including the UPS, are declining with age (21). Therefore, it seems plausible to draw a link between the two.

In this review, we aim to provide an overview of the evidence that led the majority of the scientific community to adopt the toxic protein aggregates theory, and how these findings keep pointing out to dysregulation of the machineries responsible for PQC. We shall present recent discoveries and advancements in the field, from the molecular level to promising therapeutics approaches; while also pointing out to some substantial challenges, unsolved knowledge gaps, failed trials, and a turmoil concerning suspicions for scientific misconduct, all of which are still casting some doubts and skepticism concerning the currently ruling dogma.

## Dysregulation of the UPS in human pathophysiology

The UPS is involved in nearly all cellular processes, and it is therefore not surprising that its inadequate function can lead to pathological conditions. Taking into account the number of E3 ligases, DUBs, and all other components of the ubiquitin system, the UPS encompasses 6–8% of the encoding human genome. This underscores the fundamental role of the UPS in cellular function, and the relatively common involvement of its components in human diseases (22). Probably, the most extensively studied field with regard to the UPS is cancer, and specific E3 ligases were shown to underlie various types of tumors: MDM2 (23), the E3 ligase of p53 – in sarcoma; pVHL (24), the ligase of HIF1α – in virtually all cases of clear cell renal cell carcinoma; and BRCA1 (25), known throughout the public to be involved in breast, ovarian, and pancreatic carcinoma – are only a few examples.

Given its central role in protein turnover, alongside the proteinopathic nature of NDD, the UPS has been extensively studied for its possible involvement in the pathogenesis of NDD, as well as a potential platform for drug development (26). The tight relationship between PQC and NDD is further illustrated by some of the models used to study these diseases. For instance, it was reported that impairing UPS function via the knockout (KO) of a single 19S proteasome subunit in motor neurons, replicates ALS in a mouse model. The authors reported this KO to result in accumulation and altered localization of NDD-associated proteins (TDP-43 and FUS), shuttle proteins (Ubiquilin2 and Optineurin), ubiquitin, and the 19S proteasome (27).

## Protein aggregates in neural disease

The fact that neurons are largely non-dividing cells, alongside their distinctive metabolic nature, projects also on the orchestrated turnover of their proteins and render them particularly vulnerable to toxic protein aggregation (28). Early links between accumulated proteins and NDD pathogenesis originated from reports concerning the observation of protein aggregates in Alzheimer’s, Parkinson’s, Huntington’s, and other diseases (29, 30). In a paper from 2006 – retracted in June 2024 – amyloid-β was suggested to accumulate extracellularly in the brain, thereby leading to memory deficits. To a large extent, this report served as the basis for numerous follow-up studies, and to further strengthen the leading hypothesis for the pathogenesis of Alzheimer’s, according to which the critical event underlying the initiation of the disease is the extracellular deposition of amyloid plaques.

**Figure 2:**
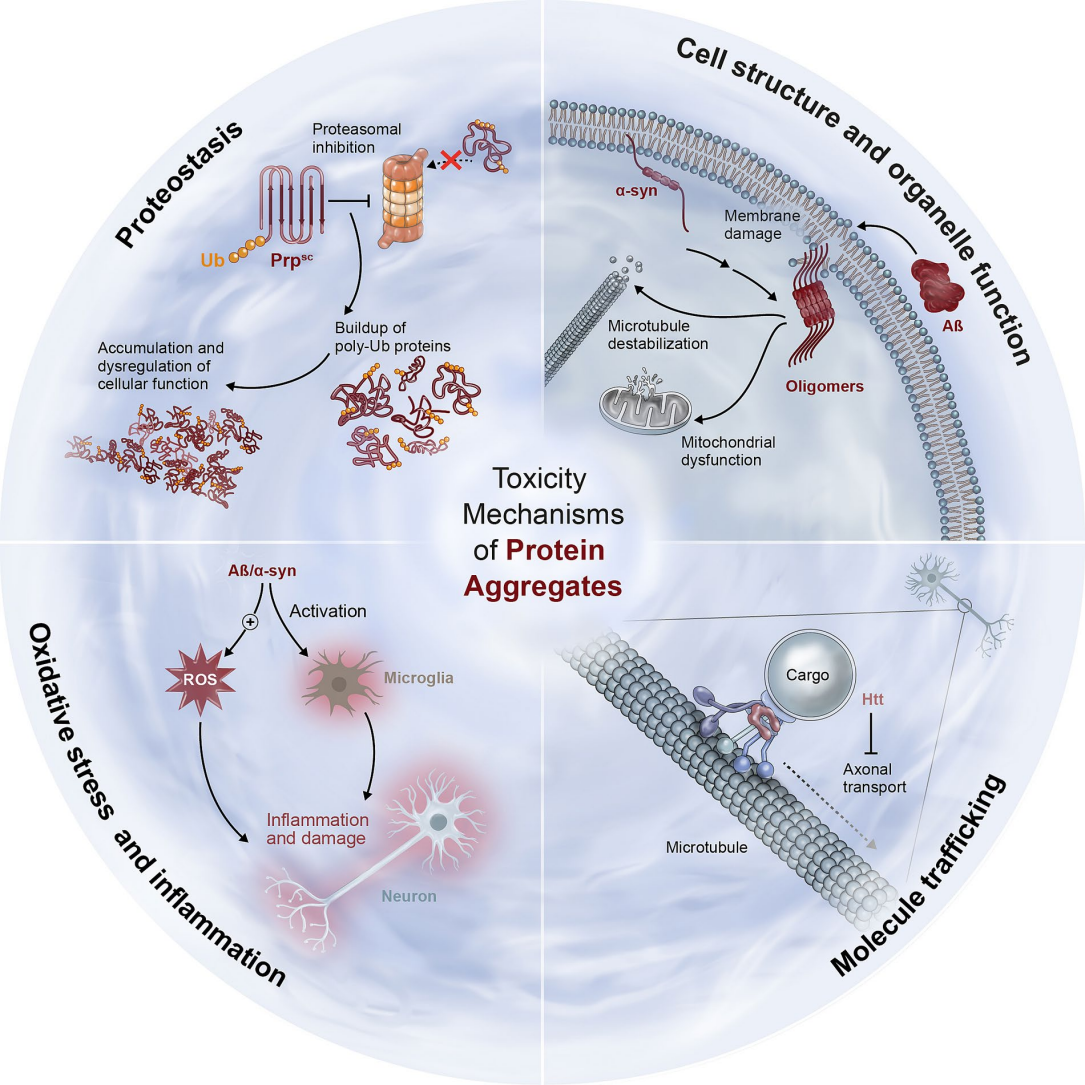
Mechanisms for neuronal toxicity caused by aggregated proteins in neurodegenerative diseases. NDD-related aggregated proteins exert neurotoxicity via different mechanism, including impairment of adequate proteostasis, damage to cellular structures, inhibition of molecule trafficking along axons, and neural injury due to oxidative stress and inflammation. See Table 1 and text for further details and references.

An extensive share of research into NDDs is concerned with the toxicity mechanism(s) of NDD-associated proteins (Table 1; Figure 2). Aggregates of unfolded proteins were shown to affect also unrelated proteins, via their binding and subsequent sequestration, leading to their effective depletion and loss of their function (31). Misfolded protein aggregates were also shown to stimulate ER-associated stress, the continuous activation of which is leading to apoptosis (32). Additional damage mechanisms include the disruption of organelle membranes and interfering with their adequate functions (33, 34), as well as the disruption of nuclear integrity and trafficking, which are important for normal cell functioning (35, 36). Interestingly, members of the nuclear pore complex, implicated in NDD as well as other diseases (37, 38), were also linked to PQC via their effect on proteasome localization (18). Some aggregated proteins were also suggested to cause neuroinflammation and oxidative stress, thereby aggravating the neural damage (39, 40).

**Table 1:** Neurotoxic effects of NDD-related protein aggregates.

Function	Toxicity mechanism	Examples	Ref
Proteostasis	Decreased chaperone activity, UPS and autophagy impairment	PrP can block the entry of other proteins into the proteasome; htt can lead to autophagic dysfunction, and α-synuclein can interfere with CMA	(31, 45, 52–54)
Cell structure and organelle function	Microtubule destabilization, nuclear dysfunction, membrane disruption and subsequent electrolyte imbalance	Amyloid-β and α-synuclein can disrupt membranes and function of mitochondria	(33, 34, 121, 122)
Molecule trafficking	Nucleocytoplasmic and axonal transports	Htt can impair both nucleocytoplasmic and axonal transport, the latter may be compromised also by SOD1	(35, 123, 124)
Oxidative stress and inflammation	Generation of free radicals, metal binding, and microglia activation	Amyloid-β, tau, and α-synuclein aggregates can lead to ROS production, and aggravate neuroinflammation	(39, 40)

Notably, toxic protein depositions are observed also in other medical conditions – and in tissues other than the brain. Amyloidosis, referring to the abnormal protein deposits, mostly extracellularly, is a group of pathological conditions caused by such aggregates, either directly or indirectly. Categorically, amyloidosis is classified into AL amyloidosis, caused by aggregated light chains; TTR amyloidosis, due to precipitation of transthyretin (pre-albumin); and AA amyloidosis, stemming from serum amyloid A in chronic inflammatory diseases (41, 42). Unlike NDD, the culprit proteins in these conditions are derived from cells other than the ones damaged (e.g., AL amyloid from plasma cells, and TTR amyloid synthesized by the liver – both can affect the heart). Also, in the case of hypertrophic cardiomyopathy – defined by the pathologic accumulation of myocardial contractile proteins (43), these proteins are not by themselves damaged. The sequelae of the disease stem from the effect of the hypertrophied myocardium on cardiovascular function, rather than a cytotoxic effect of the accumulating proteins, as seen in NDD. Interestingly, in one of these cases, it has been suggested that the UPS is involved in the underlying pathogenesis (44). All-in-all, toxic protein aggregates, in the CNS as well as in other tissues, may be both directly and indirectly linked to the UPS.

## Parkinson’s disease: various errors, similar consequences

Intriguingly, Parkinson’s Disease provides an example for each of the above cases: *α*-synuclein, found in cytoplasmic inclusions called Lewy Bodies, may be pathogenic due to mutations in (or amplification of) its encoding gene SCNA. In other patients, the observed *α*-synuclein inclusions may stem from genetic alterations in the Leucine-rich repeat kinase 2 (LRRK2), the linkage of which to α-synuclein is yet to be fully unraveled. It has been suggested that both proteins are relying on CMA, at least in part, for their degradation, and that pathogenic mutation in LRRK2 may jam CMA at large, leading to α-synuclein accumulation (45–47). This possibility portrays yet another possible passage for aggregate formation, in which neither the toxic aggregated protein is abnormal to begin with, nor are the selective components responsible for its targeting for proteasomal or autophagic degradation (e.g., E3 ligases or shuttle proteins). In this case, it is rather an unrelated dysfunction of the proteolytic machineries that is causing the accumulation and toxic aggregation of the culprit protein.

This raises broader questions concerning the identity of the proteins causing NDD, and the variety of mechanisms underlying their aggregation. For instance, why *α*-synuclein, but not other proteins, is the one to stand out in the crowd of thousands by having a pathogenic effect due to a general malfunction in proteostasis. It seems like an unlikely coincidence that the wild type α-synuclein becomes pathogenic in face of dysregulated CMA, while the mutated α-synuclein causes the same disease in familial cases. These observations suggest that numerous factors underlie the observation that some proteins are prone for creating toxic aggregates, while other do not, some of which reside beyond the scope of PQC. For instance, the propensity of a given protein to form insoluble aggregates with adverse cellular outcomes due to its inadequate removal, may determine whether it will become toxic and disease-causing factor. While the biophysical aspects of aggregate formation, as well as other determinants of the harmful potential of different accumulating proteins in neurons may provide important insights into NDDs, we shall keep our focus mainly on questions concerned with protein degradation.

Another inherited form of Parkinson’s disease involves a mutation in the E3 ubiquitin ligase Parkin. The ligase is involved in proteasomal degradation of misfolded and aggregated proteins, as well as mitophagy – autophagic degradation of mitochondria. While the direct link between Parkin and *α*-synuclein is yet to be fully understood, in Parkinson’s patients harboring inactivating mutations in the former, aggregates of the latter can be found in neurons (48). Pathogenic mutations in the PRKN gene lead to dysregulation of mitochondrial turnover in neurons (49) as well as defects in synaptic endocytosis, the inadequate function of which was suggested to lead to dopamine accumulation and subsequent toxicity to dopaminergic neurons, which is a hallmark of Parkinson’s Disease (50).

Mutations in a variety of UPS components, mainly E3 ubiquitin ligases and DUBs, were shown to underlie the pathogenesis of different NDDs (see below). Such alterations are thought to lead to dysfunction of their role in UPS-mediated protein removal, and therefore to protein aggregates. Nevertheless, in many of these cases the exact mechanistic link between the mutated UPS component and the neurotoxic aggregated proteins is yet to be discovered. This molecular task seems particularly complex in NDD, given that even our clinical understanding and classification of these conditions is still developing. An intriguing example for our limited understanding of an NDD is presented by cases of Parkinson’s Disease associated with Parkin mutations: Lewy bodies, considered a hallmark of Parkinson’s, are round-to-elongated eosinophilic cytoplasmic inclusions, characterized by a dense central core composed by tightly packed *α*-synuclein filaments, and a surrounding pale rim with more loosely arranged α-synuclein. While different types of α-synuclein aggregates are a common finding (synuclein inclusions), the hallmark Lewy Bodies are absent in many cases of the disease. Further, in some cases of Parkinson’s Disease, no protein aggregates are found (51). Even in Parkinson’s Disease – one of the most extensively studied NDD – we are only now beginning the required phenotypic stratification of patients – one that may ultimately be used for a proper and more precise treatment.

Much of the research regarding PQC in NDD is focused on ways through which impaired protein degradation may lead to the accumulation of neurotoxic proteins. Interestingly, other studies have reported a reciprocal effect, i.e., impairment to the function of the UPS and autophagy by aggregated proteins. Therefore, while a specific protein may be recognized as the alleged culprit, it was suggested that the effect of aggregated proteins such as Huntingtin may be toxic, at least in part, via inhibition of protein removal and the subsequent accumulation of misfolded proteins (31, 45, 52–54). This scenario raises the possibility that in some cases, the cause and effect may be opposite to the previously suggested mechanisms, i.e., abnormal degradation leading to the accumulation of a discrete protein, or that the two phenomena may constitute a self-aggravating vicious cycle. In any case, the broad effect exerted on proteostasis and protein misfolding by alleged NDD-associated raise questions on whether the toxicity of such proteins is inherent, mediated via general dysfunction of PQC, or both. Besides for the mechanistic understanding of Parkinson’s pathophysiology, better understanding of the causative agent(s) in different subtypes of the disease may also provide better prognostic and therapeutic approaches.

## ALS and FTLD – common errors, variable consequences

Considering the different phenotypes presented by NDDs from an anatomic point of view, one may point out to the different functions provided by the different brain regions to explain the clinical landscape of each NDD. Yet, even if certain genetic alterations are most apparent phenotypically in a specific brain region (e.g., *α*-synuclein and the substantia nigra in Parkinson’s disease), there are still cases in which it is not entirely understood how the same NDD-associated protein, or the same genetic mutation, are affecting regions of the central nervous system that seem unrelated, thereby leading to distinct NDDs. For instance, tau can be present in various types of NDDs (i.e., tauopathies), such as Alzheimer’s Disease, frontotemporal lobar degeneration (FTLD, formerly referred to as frontotemporal dementia – FTD), progressive supranuclear palsy (PSP), Parkinson’s Disease, and corticobasal degeneration (CBD) (55). Similarly, *α*-synuclein can be found in Parkinson’s disease as well as in multiple system atrophy (MSA) (56).

In both ALS and FTLD, inclusions may contain either TAR DNA-binding protein 43 (TDP-43) or fused in sarcoma (FUS), while superoxide dismutase 1 (SOD1) is another culprit reported in ALS, and tau aggregates were identified in FTLD. FTLD-tau cases are distinctive from Alzheimer’s Disease as they lack extracellular amyloid-β plaques. Tau aggregation may stem from mutations in the encoding MAPT gene in familial cases, and can also be sporadic. Interestingly, MAPT mutations do not give rise to Alzheimer’s Disease, underscoring the preceding role of amyloid-β deposition in the pathogenesis of Alzheimer’s. With regard to FTLD-TDP-43, the most common underlying genetic alteration is localized to a gene other than that of the culprit protein – C9orf72, encoding a protein of unknown function which was not found to aggregate. Mutations in C9orf72 also account for a large fraction of familial ALS cases, as well as some sporadic ones, and can simultaneously give rise to ALS and FTLD. Less commonly, mutations in TAR DNA-binding protein (TARDBP), the gene encoding TDP-43 which is associated with RNA processing function, are accounted for the either FTLD or ALS. Both gain of function and loss of function mechanisms of pathogenesis were documented. Intriguingly, some forms of FTLD show no protein inclusions, which further underscore our gap of knowledge concerning this condition in specific, as well as NDDs in general (57).

While many of the above mutated proteins involved in pathogenesis of the two diseases are not inherent components of PQC machineries, some other cases were linked with dysfunction of autophagy or the UPS more directly. Optineurin, a shuttle protein targeting proteins for autophagic degradation, was implicated in familial cases of ALS, where affected individuals harbor mutations in its encoding OPTN gene. Similarly, mutations in the E3 ubiquitin ligase VCP (Valosin-containing protein), a key component of ER-associated degradation of misfolded proteins, were suggested to contribute to the pathogenesis in FTLD and multisystem proteinopathy (58). While genomic and transcriptomic studies have revealed these and other alterations in NDD, their contribution to disease pathogenesis is yet to be unraveled.

## Alzheimer’s disease: a tale of two culprits, aging, and some other risk factors

Alzheimer’s Disease, the most commonly diagnosed NDD and probably the most extensively studied one, is characterized by both extra- and intra-cellular protein deposition. Extracellular deposition of amyloid-β in amyloid plaques is considered to be the fundamental event that initiates the development of the disease. The second hallmark of Alzheimer’s, believed to follow the formation of amyloid plaques, is the intracellular aggregation of the protein tau in neurofibrillary tangles. In agreement with the extracellular cleavage of the amyloid-precursor protein (APP) and deposition of amyloid-β plaques, both normal and pathogenic cleavages are carried out by extracellular secretases. While *α*- and *γ*-secretases – in concerted activities – yield a soluble and harmless peptide, cleavage of APP by the β- and γ-secretases generates an amyloidogenic peptide (43, 59). In the majority of familial, early-onset Alzheimer’s, mutations were identified in the genes encoding subunits of the γ-secretase complex – PSEN1 and PSEN2, encoding Presenilin-1 and Presenilin-2, respectively. These mutations result in a gain of function, leading to excessive production of the amyloidogenic extracellular peptide (60).

While the above proteolytic activity is not directly related to the UPS or autophagy, the UPS was implied in some cases of Alzheimer’s Disease through a frameshift in one of the genes encoding for ubiquitin – UBB (61).

This frameshift results in a ubiquitin molecule that can undergo poly-ubiquitination, forming ubiquitin chains that are resistant to deubiquitination and subsequently to the inhibition of UPS activity (62). This alteration, marked UBB + 1, was suggested to be sufficient to initiate the cascade of events leading to amyloid-β plaque deposition and tau tangles formation (63). While it is still unclear how this mutation is leading to the aggregation of proteins, this observation raises some intriguing questions. Since UBB is only one out of 4 genes encoding ubiquitin, this alteration, if indeed pathogenic, is more likely to exert its effect via a gain of function, rather than loss of function mechanism. If that is the case, and given the universality of ubiquitin’s role throughout all types of tissues, how likely it is that the deleterious effect of UBB + 1 is restricted only to the entorhinal cortex, for instance, while the rest of the body is spared? Similarly universal – mutations in the UBA1 gene, encoding for the E1 ubiquitin activating enzyme, were identified in X-linked spinal muscular atrophy and a rare autoinflammatory syndrome (64, 65). While a selective effect of a dysfunctional UBA1 may be potentially explained by the presence of UBA6, a second E1, ubiquitin-activating enzyme, it is unclear if UBA6 actually serves as an alternative for the canonical E1, UBA1 (66). It remains to be determined whether these subtypes of the disease share common mechanistic basis – both between themselves and with the more common senile type. Hopefully, better understanding of the unique genetic landscape of less common subtypes may also provide important insights concerning Alzheimer’s in general.

## Prey or predator: who is to blame when PQC goes awry?

From biochemical and cellular points of view, the observed inadequate removal of proteins may stem mostly from two origins – the substrate protein itself, or the machinery responsible for its removal. In the first case, the substrate protein may be genetically altered (or amplified) in a way which prevents its normal removal (or overwhelms its degradation machinery), thereby leading to its accumulation and subsequent aggregation. In the second case, the degradation machinery may be dysfunctional, and a protein which is naturally prone to misfolding and aggregation, is therefore accumulated. Either way, the above steps are likely propelling one another, generating a vicious cycle which leads to aggregates formation. In yet another scenario, an aggregated protein may by itself be intact, functional, and otherwise normal – while the mechanism responsible for its degradation (or folding) is abnormal and/or dysfunctional. This scenario provides an explanation for a common observation in NDD, according to which the aggregated protein, while toxic and may be held as culprit, shows no genetic mutation, amplification, or inherent structural aberration. This is true with regard to *α*-synuclein in some cases of Parkinson’s disease. Importantly, besides its mutation, being an intrinsically disordered protein may explain, at least in part, the propensity of the WT protein to form aggregates (67). Some NDD-related protein and their suggested link to PQC are presented in Table 2.

**Table 2:** Dysregulation of PQC in representative NDDs.

Disease	Protein	PQC linkage	Altered genes	Gross CNS changes	Phenotype	Ref.
Alzheimer’s	Amyloid-β, tau	Mutated ubiquitin gene – UBB	APP, PS-I, PS-2, Trisomy 21	Cortical atrophy – mostly frontal, temporal, and parietal lobes	Dementia	(63)
Parkinson’s	α-synuclein, tau, none	Mutated E3 ubiquitin ligase – Parkin; CMA inhibition by α-synuclein	α-synuclein mutations or amplification, LRRK2, DJ-I, PINKI, Parkin	Pallor of substantia nigra	Hypokinesia ± dementia	(125)
Amyotrophic lateral sclerosis	SOD1, TDP-43, FUS	Mutated SQSTM1/p62 and E3 ubiquitin ligase – VCP	SOD1, TDP-43, FUS, C9orf72	Motor neuron loss (anterior horn, spine) +/− atrophic precentral motor gyrus (cortex)	Weakness/ paralysis	(58)
Frontotemporal lobar degeneration	Tau, TDP-43, FUS		Tau, TDP-43, FUS, Progranulin, C9orf72	Atrophic frontal and temporal lobes	Behavioral and language disturbances	
Huntington’s	Huntingtin	Htt impairs autophagic protein removal; SQSTM1/p62 is implied in htt aggregation	Htt	Atrophic caudate and putamen	Hyperkinesia	(53, 89)
Creutzfeldt-Jakob	PrP	Misfolded PrP impairs proteasome function	PrP^SC^	Spongiform transformation of cerebral cortex, caudate, putamen	Rapidly progressing dementia	(52)

## Compartmentation of the proteolytic machinery: concentrating the UPS in membrane-less organelles

While NDDs underscore the challenge presented by different pathological conditions affecting different brain regions, studies from recent years show that the “localization of events” is important also at the molecular level. Membrane-less organelles (MO), also referred to as liquid–liquid phase separation (LLPS) condensates, present a paradigm shift from the classical theory of cell organization into membrane-bound organelles, as well as to the spatial organization of proteolysis (68). Importantly, the relationship between MOs and cellular proteolytic system is bidirectional. Alterations in the local concentration of proteins that play key role in condensates’ regulation, via their degradation, among other mechanisms, may affect both the formation and properties of LLPS condensates (69). In agreement with their unique boundaries dictated by biophysical forces rather than a physical barrier, MOs are relatively transient structures. Such partitions, requiring no structural formation, can nevertheless facilitate the proximity required for efficient execution of different cellular processes. While much of the early studies into MOs identified RNA and RNA-binding proteins, suggesting “local concentration” serving transcription and/or RNA metabolism regulation, recent reports point MOs can also facilitate protein degradation. Probably due to the unique requirement for locally regulated PQC within their different extensions, MOs were shown to play a key role specifically in neurons (70–72). NDD-associated proteins such as TDP-43 and FUS were also suggested to reside in MOs, therefore implying the involvement of these condensates in NDD (73).

With regard to NDD, aberrant MO function in synapses and axons was suggested to contribute to the pathogenesis in ALS (74, 75). The protein FUS was shown to form liquid compartments, which can transform into aberrant aggregates in a manner that is accelerated by the presence of mutations found in ALS patients (76). In the related FTLD, mutated TIA1 was suggested to promote phase separation, but in the expense of their dynamic nature. It was suggested that the decreased condensates’ dynamics attract TDP-43, rendering it immobile and insoluble (77). Intriguingly, concentration of TDP-43 in stress granules was recently shown to serve as the initiating step of pathological aggregate formation in NDDs such as ALS and FTLD (78). TDP-43 was suggested to aggregate in complex LLPS droplets termed anisosomes, into which it is chaperoned by HSP70 (79). Genetically altered C9orf72 (i.e., hexanucleotide expansion), the most common cause of ALS and FTLD, was suggested to induce anisosome formation and subsequent pathological TDP-43 aggregation in the nucleus, despite the lack of TDP-43 mutations (80). Importantly, MOs were implicated also in NDDs other than ALS and FTLD. It has been reported that the DJ-1 protein, mutations in which are associated with an autosomal-recessive form of Parkinson’s Disease, plays a role in condensates’ function in response to neurotoxic agents (81). While it is yet to be unraveled how mutations in the PARK7 gene – encoding for the DJ-1 protein – result in toxic protein aggregates, a growing body of evidence strongly suggests the involvement of MOs in NDD (82).

While the pathologic formation of protein aggregates from LLPS condensates may be the result of dysregulation of several mechanisms, the focus of this review remains the UPS, including with regard to MOs. As described above, targeting proteins for degradation is an elaborate cascade of concerted events, some of which are reversible. While in most cases UPS components are soluble and are not constitutively bound to a specific organelle, it may seem inefficient for such an operation to be executed by mediators that have no spatial association to one another. It would seem more reasonable for the UPS to bring into proximity the various enzymes and other components required for protein removal. Indeed, ubiquitin, specific E2 carriers, E3 ligases, DUBs, shuttling proteins, as well as the proteasome were reported to localize into MOs, facilitating the removal of specific substrates (83).

## Monogenic, young, anticipated: Huntington’s disease as a prototypic poly-Q disease

Huntington’s Disease is an autosomal dominant disease caused by expansion of tri-nucleotide repeats (CAG) in the N-terminal domain of the huntingtin (htt) gene, encoding the amino acid Q (glutamine). The resulting degeneration is localized to striatal neurons, leading to hyperkinetic movement disorder and dementia. Intriguingly, htt expression is not limited to the brain, but rather seen throughout all the body’s tissues – with no apparent consequences. It is still unclear why the deleterious effect of the pathogenic htt is restricted only to the brain (84, 85). The expansion of CAG repeats is exacerbated during spermatogenesis, a process termed anticipation (86). While the normal number of CAG repeats is 6–35, the number of repeats within the pathogenic range is also important, with longer expansions correlating with severity of the disease as well as a younger age of its presentation (43, 85). Interestingly, intracellular htt inclusions were suggested to play a mitigating role in the affected striatal neurons, as htt aggregates are sequestered from the major cellular machineries (87).

While the genotype underlying poly-Q diseases is known, and there are no sporadic cases of Huntington’s caused by other defects, including in components of the UPS, the relationship between PQC machineries and the culprit protein is nevertheless of great interest, as it may provide the means to alleviate the neurotoxic effect of htt. In fact, while the mechanism(s) of toxicity exerted by abnormal Poly-Q proteins is unclear, the presence of htt inclusions was suggested to represent a protective sequestration of the protein which subsequently restricts also its toxic effect(s). The observation that htt inclusions may in fact serve as a protective mechanism, alongside the finding that ubiquitin, proteasome subunits, and other component of the system were found within htt inclusions (88), supports the notion that the UPS is playing a protective role in Huntington’s Disease. The shuttle protein p62, also found in htt-positive inclusions, was shown to be more abundant in affected cells, pointing to a regulatory mechanism which upregulates its expression, probably as part of htt targeting into aggregates (89). These findings focus on the mechanism(s) responsible for htt trafficking to aggregates, and may be perceived as a “last stop” for mutated htt molecules marked by ubiquitination. Notwithstanding, evidence suggests that htt ubiquitination is reversible within inclusions via the activity of DUBs, underscoring the dynamic nature of ubiquitination also after htt was successfully targeted to the aggregates (90). This observation strongly suggests that inclusion bodies may serve more than just “trash collection sites,” and are rather active protein degradation centers. Since protein aggregates are sometimes considered as irreversible depositions which cannot be dismantled by the UPS or autophagy, it is conceivable that therapeutic approaches targeting the NDD-associated protein may only stop further progression of the disease, but not reverse it. If htt inclusion bodies are indeed sites for dynamic, ‘concentrated’ activity of the UPS, inhibition of additional htt accumulation may allow the removal of exiting deposits. Furthermore, if htt (or other) protein aggregates are active degradation foci rather than “end point” precipitation sites – it means that their catalytic activity may be further enhanced. These potential clinical implications underscore the importance of better understanding the composition, catalytic activities, and regulatory mechanisms of NDD-associated inclusion bodies, even in cases where the UPS is not part of the underlying pathogenesis, but rather a potential solution. Recent studies suggest that the unstable alleles containing CAG repeats undergo somatic expansion over a few decades. This phenomenon may explain the onset of clinical manifestation in Huntington’s patients, which is typically observed during the 4th–5th decades of life (91, 92).

## Infectious toxic proteins: a unique case – or a case study?

Prion disease, historically referring mostly to Creutzfeldt-Jakob Disease (CJD), is an example of NDD in which the identity of the pathogenic protein is known, and its mechanism of toxicity is quite established. PrP, a cytoplasmic protein with no currently known function, undergoes a conformational change from *α*-helix (PrP^C^) to β-pleated sheath (PrP^SC^) – a form that is resistant to proteolytic digestion. A key feature of prion disease is the fact that PrP^SC^ causes the conversion of additional PrP^C^ molecules into the abnormal PrP^SC^. The initial conformational alteration may happen spontaneously (in sporadic CJD), or as a result of an inherited mutation in the encoding PRNP gene (familial CJD, fatal familial insomnia). The initial event may be the result of an exposure to exogenous PrP^SC^ (Variant CJD) which can be derived from digested meat of an animal affected by bovine spongiform encephalopathy, blood transfusions, and transplanted organs from donors harboring the pathogenic prion (93).

Unlike most NDDs, CJD is a rapidly progressing condition, with mean survival of only 7 months from diagnosis (44, 93). While this aggressive course of CJD may be attributed to its infectious nature, it may also stem from the mechanism of toxicity caused by the culprit proteins in different types of NDD. One may suggest that the more inherently toxic, the faster a given NDD-associated protein will induce neuronal damage and clinical manifestation. In contrast, NDD-associated aggregated proteins which exert their toxicity via impairment of PQC, leading to gradual accumulation of unfolded proteins, may require longer periods to cause a substantial damage, and are characterized by a smoldering clinical course. This hypothesis may be challenged by some evidence suggesting that many of the NDD-associated proteins are in fact “infectious” in a prion-like fashion. Notably, the “gut-brain” hypothesis in Parkinson’s Disease, according to which Lewy Bodies are initially formed in the enteric nervous system, and spread via the vagus nerve into the CNS, is an example for the spreading of pathogenic inclusions (94). The prion-like hypothesis, postulating that most (if not all) of the proteinopathies may be spreading via an infectious mechanism, is supported by additional studies that have identified additional NDD-associated proteins, including *α*-synuclein, tau and amyloid-β, which also demonstrate prion-like infectious and spreading (95–98). While the extent of the clinical relevance of these findings in human patients affected by NDDs such as Alzheimer’s is a subject for further investigation, some types of NDDs may suggest that an infectious form, besides for the culprit proteins involved, may be part of the distinction between different conditions. Taken together, the phenomenon of prions underscores that intact proteolytic machineries may not suffice in order to maintain adequate PQC, and that substrates resistant to degradation may have deleterious effects on protein turnover in general.

ALS-PDC (Parkinsonism dementia complex) is a rare NDD endemic to Guam, in which amyloid-β plaques, tau tangles, and *α*-synuclein inclusions can be found in the brain of affected individuals. Furthermore, amyloid-β and tau were suggested to present prion characteristics, rendering this condition as a double prion disease. The molecular phenotype of the two proteins was shown to differ from that demonstrated for the amyloid-β and tau in the case of Alzheimer’s Disease (99). These findings underscore the complexity of NDD landscape, where the identity of the culprit protein(s) by itself may not necessarily be deterministic with regard to the actual type of the NDD inflicted by it.

## Defective components of the UPS as underlying cause for neurodevelopmental disorders

Improper function of the UPS was demonstrated to underlie not only diseases stemming from toxic aggregation of proteins, such as NDD, but also in neurodevelopmental diseases. In such conditions, presenting much earlier than NDD and largely lack the hallmark of protein aggregates, single mutations in either an E3 ubiquitin ligase or a DUB were suggested to account for different neurodevelopmental syndromes (100, 101).

An illustrating example of the linkage between the UPS and neurodevelopmental diseases is Angelman Syndrome. In this severe disorder, characterized by ataxia and intellectual disability, among other neurological symptoms, the E3 ubiquitin ligase UBE3A/E6AP (E6-associate protein) is nonfunctional due to mutations or deletions in the maternal allele, and concomitant epigenetic silencing of the paternal allele (i.e., parental imprinting) (102–105). UBE3A was demonstrated to play a key role in synapse formation, which may account for the cognitive impairment observed in Angelman Syndrome, at least in part (106).

The pathogenesis of most of these conditions is postulated to derive from inadequate neurogenesis, with emphasis on dysregulated synapse formation and plasticity. Such deleterious effects on neural development were suggested to arise from the critical role the UPS plays in axonal and dendritic development, as well as synapse formation and maintenance. It was shown that in neurons, compartmentation of PQC is especially crucial, and the UPS fulfills specific roles in the different processes of neurons, thereby contributing to the proper function and maintenance of each of them (107). Intriguingly, recent studies showed that also the proteasome is regulated by its compartmentation across different cell regions (18, 108), and its shuttling was described also along axons, as part of the mechanism underlying synaptic development (109).

While adequate protein degradation was directly linked to neurodevelopmental disorders, recent work suggests that brain malformations manifested as seizures, cognitive impairments, and other disabilities, can derive also from improper function of protein folding machineries. It was reported that such conditions may stem from mutations in subunits of the T-complex protein-1 ring complex (TRiC) chaperonin, responsible for co- and post-translational folding of 5–10% of the proteome (110–114). While the described pathogenic alterations do not affect directly components of the UPS or autophagy, the above findings raise questions regarding the ability (or failure) of protein removal machineries to degrade the growing pool of unfolded proteins. Furthermore, TRiC was shown to facilitate the folding of aggregate-prone proteins, as well as key mediators of essential cell functions such as actions, DNA/RNA replication factors, protein kinases including subunits of the mTOR complex (101), as well as NDD-related proteins such as huntingtin (115, 116). Further research is required in order to unravel whether the phenotypes caused by TRiC mutations are the result of protein aggregates, dysfunction of cellular pathways dependent on the unfolded proteins, or both.

## Discussion

### High hurdles in the route towards better understanding NDD

The fact NDD affects non-dividing cells impose a great challenge on studying these pathologies, as well as predisposing cellular conditions. The use of cell lines is virtually impractical. Furthermore, due to lack of mechanistic understanding of most NDDs, any model aiming to recapitulate certain such disease is limited to phenotype(s) and biomarker(s) that do not necessarily bear a causative role. Therefore, while some traits of NDD in a given model may faithfully mimic those observed in human patients, they may arise due to different reasons, or have different effects. One cannot be certain that a given animal model which is used to study a human NDD and shares identical mutation with human patients, also shares common pathophysiology and underlying mechanisms. In fact, even different NDDs can share the same “hallmark” protein markers or pathogenic mutations, yet comprise separate entities.

For instance, why do cytoplasmic inclusions of *α*-synuclein or Lewy Bodies, exert their most prominent effects by forming in the substantia nigra of Parkinson’s disease patients, in the cortex and brain stem of patients suffering from Dementia with Lewy Bodies, and in oligodendrocytes of individuals affected by Multiple System Atrophy? Similarly, why out of two patients presented with neural inclusions of TDP-43, one of them suffers from FTLD, while the other develops ALS due to destruction of neurons in the motor horns of the spine? And in yet another ALS patient, harboring a SOD1 or FUS mutation, cytoplasmic inclusions are in fact negative for TDP-43. Furthermore, genetic mutations underlying TDP-43 inclusions may be found in the TARDBP gene encoding this protein, but a C9orf72 mutation can also lead to such inclusions (43).

In Alzheimer’s Disease, besides for the direct cognitive sequelae of amyloid plaque formation, cerebral amyloid angiopathy can be found in virtually all brains of affected patients. This pathology of blood vessels, constituting the leading risk factor for lobar hemorrhage, can be found also in brains of patients not affected by Alzheimer’s. This raises a question concerning other factors contributing to formation of amyloid depositions and dementia in some patients, but not in others. Notably, the amyloid-β peptide characterizing the neuritic plaques in Alzheimer’s differs from the one affecting cerebral blood vessels – while the former is mainly consisted of amyloid-β_42_, the latter is mostly comprised of amyloid-β_40_. Nevertheless, this finding represents a variable predominance rather than a distinct or exclusive composition of amyloid plaques, thereby calling for further investigation into additional differences between cases of cerebral amyloid angiopathy with or without Alzheimer’s Disease.

Given our current limited understanding of the linkages between genotypes, molecular and cellular findings, affected brain regions, and clinical phenotypes – it is not clear whether we can recapitulate with sufficient certainty a specific NDD via a given model? Is it possible to reproduce the context which resulted in development of an NDD in the frontotemporal region rather than the anterior horn of the spinal cord? Some of these pitfalls are clearly valid to some extent, also in various other fields of biomedical research. Nevertheless, many of these challenges are perhaps harder to overcome in the case of NDD, and these methodological hurdles join the lower availability of human brain tissues, let alone fresh ones, compared with other areas of research (e.g., cancer). Brain biopsies, let alone for non-malignant etiologies, are less common than sampling of other tissues and pathologic processes.

Despite these and other challenges, the neuroscientific community has made significant strides in recent years. Advancements in fields such as genetic analyses and protein expression studies, as well as the growing availability of such technologies, facilitate research that will hopefully address some of the questions raised in this review along with many others. The difficulty in obtaining human brain samples is tackled by neuro-biobanks in some countries, and the different models for studying NDD, while imperfect, have yielded invaluable findings that keep advancing the field despite the aforementioned challenges. All-in-all, while studying NDDs poses some objective hurdles, these cannot slow major achievements in the field, and the years to come will surely provide further exciting discoveries.

### The toxic protein aggregates theory – challenged, yet prevailing

Recently, the theory stating that protein aggregates are the underlying cause for NDD in general, and that amyloid-β plaque formation is the critical initiating event in Alzheimer’s disease in specific, has suffered a recent blow with the retraction of a landmark research paper from 2006. Independently, some experimental Alzheimer’s drugs aiming to prevent the formation and/or remove amyloid-β plaques, such as Gantenerumab, failed in clinical trials (though later ones have shown promising results, see below). Notably, in some types of NDD it is still unclear which protein is the culprit, while in other cases more than one protein was suggested to account for the cytotoxic aggregates. As mentioned, in some NDD patients (e.g., subtypes of Parkinson’s Disease), no inclusions were found (49). Furthermore, while in some cases the aggregation of NDD-associated proteins is considered a neurotoxic event, in the case of Huntington’s disease, htt aggregation in inclusion bodies was suggested to serve a protective factor against its neuronal toxicity (88). While our understanding of the mechanisms that underlie NDD is still limited, the proteinopathic mechanism of NDDs’ pathogenesis is supported in early cases of Alzheimer’s by the effect of three monoclonal antibodies (Donanemab, Lecanemab, and Aducanumab) on the amyloid burden and clinical course of the disease (117–120). While the mechanism of action of the antibodies is mediated by engulfment of the antibody-amyloid-β complex by microglia and is not a bona fide intracellular PQC mechanism, it nevertheless underscores the role of aggregated proteins in the pathogenesis of NDD.
